# Crimean-Congo hemorrhagic fever with severe bradycardia treated with Theophylline

**DOI:** 10.1590/0037-8682-0416-2024

**Published:** 2025-06-02

**Authors:** Murat Aydın, Nurten Nur Aydın

**Affiliations:** 1 Erzurum Regional Training and Research Hospital, Infectious Diseases and Clinical Microbiology department, Erzurum, Turkey.

**Keywords:** Crimean-Congo hemorrhagic fever, Bradycardia, Cardiac complication

## Abstract

Crimean-Congo hemorrhagic fever (CCHF) is a tick-borne viral hemorrhagic fever caused by Nairovirus and characterized by fever, myalgia, arthralgia, and hemorrhagic manifestations. Severe bradycardia is a rare complication of the disease. We present a 30-year-old male patient with CCHF who developed severe bradycardia, which was successfully treated with intravenous and oral theophylline. This case highlights the need to recognize cardiac complications in CCHF and highlights the role of pharmacological interventions in improving patient outcomes.

## INTRODUCTION

Crimean-Congo hemorrhagic fever (CCHF) is an acute zoonotic viral infection caused by Nairovirus, primarily transmitted through tick bites or direct contact with infected animal tissues^1^. The disease is endemic in various regions, including the Middle East, Africa, and parts of Eastern Europe^2^. Typically, patients present with nonspecific symptoms such as fever, headache, and myalgia. However, the disease may progress to thrombocytopenia, disseminated intravascular coagulation (DIC), and hemorrhagic manifestations^3^. Cardiac complications in CCHF are uncommon, with bradycardia occurring in approximately 4% of cases^4^. Although severe bradycardia is often transient and self-limiting, it may necessitate specific pharmacological treatment^5^. 

Despite the recognition of bradycardia as a complication of CCHF, a comprehensive search of major databases, including the Cochrane Library, LILACS, SciELO, MEDLINE, PubMed, and PMC (PubMed Central), revealed no studies specifically addressing the use of theophylline in treating bradycardia associated with CCHF. This report details the clinical progression and successful treatment of a patient with CCHF who developed severe bradycardia, thereby underscoring the therapeutic potential of theophylline in such scenarios.

## CASE REPORT

A 30-year-old man was admitted to the emergency room after experiencing malaise, anorexia, fever, and diffuse muscle and joint pain for three days. Upon physical examination, he appeared lethargic yet hemodynamically stable. His vital signs included a heart rate of 88 beats per minute, blood pressure of 115/75 mmHg, and a body temperature of 38.5°C. Laboratory tests indicated leukopenia (2,750/mm³), thrombocytopenia (51,000/mm³), elevated liver enzymes (alanine aminotransferase, ALT: 177 U/L; aspartate aminotransferase, AST: 270 U/L), and elevated levels of creatine kinase (CK: 992 U/L) and lactate dehydrogenase (LDH: 720 U/L). A definitive diagnosis of CCHF was confirmed by polymerase chain reaction (PCR) testing of a blood sample.

The patient received symptomatic and supportive care, including intravenous fluids and antipyretics. Ribavirin was not administered. During the first three days of hospitalization, the patient's condition remained stable, with slight fluctuations in laboratory parameters.

**FIGURE 1:**
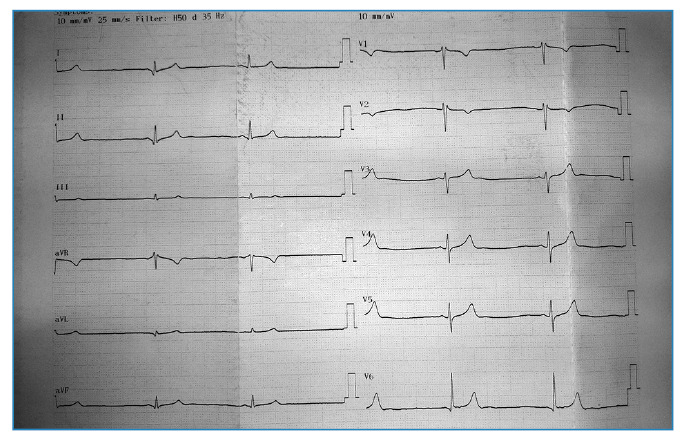
Electrocardiogram showing severe bradycardia on the fourth day of hospitalization.

**FIGURE 2:**
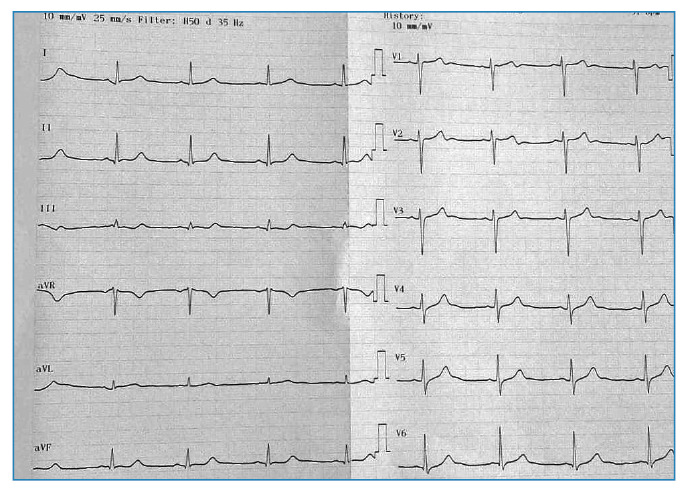
Electrocardiogram after five days of treatment with theoph.

On the fourth day, the patient reported increased fatigue and dizziness. A physical examination at this time revealed marked bradycardia with a heart rate of 35-40 beats per minute (Figure 1), although his blood pressure remained stable at 110/70 mmHg. Laboratory tests showed a further decrease in platelet count (29,000/mm³) and significantly elevated liver enzymes (ALT: 532 U/L; AST: 871 U/L) as well as persistently elevated LDH (1,092 U/L) and CK (825 U/L). Following a cardiology consultation, symptomatic bradycardia was confirmed, and intravenous theophylline (200 mg twice daily) was initiated. Over the next five days, the patient's heart rate gradually increased to 51 beats per minute (Figure 2), and he was transitioned to oral theophylline.

**FIGURE 3:**
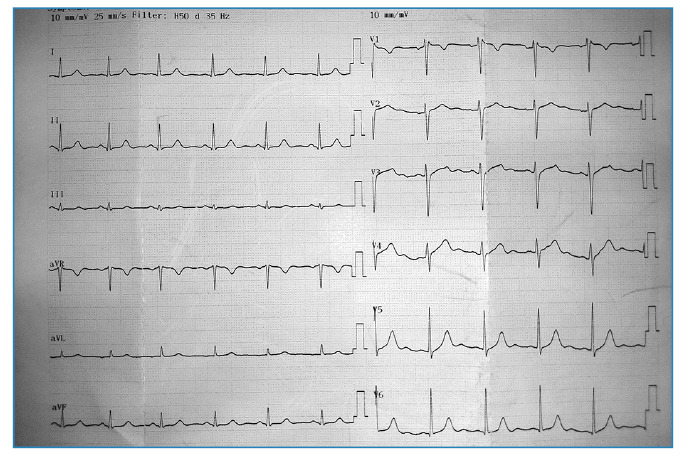
Electrocardiogram before discharge.

Subsequent laboratory tests showed normalization of platelet counts and white blood cells, reductions in ALT, AST, LDH, and CK levels, and increased heart rate to 67 beats per minute (Figure 3). The patient was discharged on the 12th day of hospitalization.

## DISCUSSION

Cardiac complications are rarely observed in CCHF, with bradycardia occurring in approximately 4% of cases^4^. Although bradycardia is often transient and benign, it can present significant clinical challenges. The precise mechanism underlying bradycardia in CCHF remains elusive, but several hypotheses exist. One proposed mechanism is viral myocarditis, which involves direct myocardial infiltration by viruses, potentially affecting electrical conduction^6^. Another hypothesis suggests that cytokine production or direct myocardial damage could be responsible^7^.

In this instance, the bradycardia was severe and necessitated pharmacological intervention. Theophylline, a methylxanthine derivative, has been effectively used to manage bradycardia. Its efficacy in this context has been corroborated by previous studies^8^ ^,^ ^9^. For this patient, intravenous theophylline led to rapid symptomatic improvement, and subsequent oral therapy facilitated further stabilization, allowing for an uneventful discharge.

Notably, Ribavirin, commonly employed as an antiviral agent in CCHF, has also been associated with bradycardia^10^. However, Ribavirin was not administered in this case, eliminating its potential contribution to the observed bradycardia.

Comparative studies of other viral hemorrhagic fevers, such as dengue fever, have also identified bradycardia as a potential complication^11^. However, the literature on this complication in CCHF is limited, predominantly describing cases that are self-limited. The severity of bradycardia observed in this patient underscores the necessity for close cardiac monitoring in individuals with CCHF, especially those exhibiting significant systemic inflammation or multisystem involvement. While severe bradycardia in CCHF is typically transient and self-resolving, pharmacological intervention may be required in certain cases to prevent adverse outcomes.

This case highlights the importance of early detection and targeted pharmacological management. The successful use of theophylline in this patient demonstrates its potential as a valuable treatment option for severe bradycardia in infectious diseases. Further research is needed to better understand the pathophysiology and to optimize the treatment of cardiac complications in CCHF.
